# Complete Genome Sequences of Four Novel Human Gammapapillomavirus Types, HPV-219, HPV-220, HPV-221, and HPV-222, Isolated from Penile Skin Swabs from South African Men

**DOI:** 10.1128/genomeA.00584-18

**Published:** 2018-06-21

**Authors:** Alltalents T. Murahwa, Tracy L. Meiring, Zizipho Z. A. Mbulawa, Anna-Lise Williamson

**Affiliations:** a Division of Medical Virology, Department of Pathology and Institute of Infectious Diseases & Molecular Medicine, Faculty of Health Sciences, University of Cape Town, Cape Town, South Africa; b Center for HIV and STIs, National Institute for Communicable Disease, National Health Laboratory Service, Johannesburg, South Africa; c SAMRC Gynaecological Cancer Research Centre, Faculty of Health Sciences, University of Cape Town, Cape Town, South Africa

## Abstract

Four novel human gammapapillomaviruses were characterized from penile specimens using genome amplification, cloning, and sequencing. The HPV-219 L1 gene showed 87% nucleotide identity to that of HPV-213 of species gamma-13, HPV-220 had 72% identity to L1 of HPV-212 (gamma-17), HPV-221 had 80% identity to L1 of HPV-142 (gamma-10), and HPV-222 had 73% nucleotide identity to L1 of HPV-162 (gamma-19).

## GENOME ANNOUNCEMENT

Human papillomaviruses (HPVs) are small nonenveloped DNA viruses of the Papillomaviridae family that infect mucosal and cutaneous epithelia (1, 2). The genus Gammapapillomavirus is the most divergent and rapidly growing genus of the family, with 27 species and 98 officially recognized genotypes (3). Here, we describe the characterization of four novel gammapapillomaviruses initially discovered using deep sequencing of the HPV L1 FAP amplicon region (4).

The penile swab collection and DNA extraction procedures have been described previously (5). The full genomes were amplified as single amplicons with back-to-back primers based on the FAP amplicon sequence and the LongRange HotStart PCR kit (Kapa Biosystems, USA). PCR products cloned into the TOPO XL vector (Thermo Fisher, USA) were sequenced on the Illumina MiSeq (2 × 300 bp) by Macrogen, Inc. (South Korea). Genome assembly was done using the *de novo* assembly function in CLC Genomics Workbench (GW) version 8.5.1 (Qiagen, USA). Splice site prediction was carried out as outlined by Van Doorslaer et al. (6). Reference clones of HPV-219 (7,108 bp), HPV-220 (7,381 bp), HPV-221 (7,326 bp), and HPV-222 (7,275 bp) were sent to the International HPV Reference Center (http://www.nordicehealth.se/hpvcenter/reference_clones/) for confirmation and assignment of type numbers.

HPV-219 is phylogenetically most closely related to HPV-213 of the gamma-13 species, sharing 87% identity in the L1 gene, HPV-220 is most closely related to HPV-212 (72% L1 identity) of gamma-17, HPV-221 is most closely related to HPV-142 (80% L1 identity) of gamma-10, and HPV-222 is most closely related to HPV-162 (73% L1 identity) of gamma-19. These HPV genotypes share <90% identity in the L1 gene (1); therefore, all four viruses are novel genotypes. The genomic organization was typical of gammapapillomaviruses, encoding five early (E1, E2, E4, E6, and E7) and two late (L1 and L2) proteins, and lacking the E5 gene. HPV-221 and HPV-222 did not have start codons for the E4 gene, but the spliced E1^E4 transcript, which encodes the primary E4 gene product (7), was identified in all four genomes.

All four viruses had a TATA box (TATAAA) (8) and palindromic E2-binding sites (ACC-N_6_-GGT) in their long control regions (9). An ATP binding site, G(x)_4_GK(T/S) (10, 11), was present in the C-terminal region of the E1 proteins of the viruses. Two conserved zinc binding domains [CxxC(x)_29_CxxC] (12) were identified in the E6 proteins and one in the E7 protein of all the viruses. HPV-219 additionally contained a putative PDZ binding domain, x(T/S)x(L/V) (13), in the E6 N-terminal region.

To conclude, we discovered four novel HPV genotypes of the Gammapapillomavirus genus. This knowledge expands the heterogeneity of the ever-growing members of the gammapapillomaviruses. The prevalence and clinical importance of these novel gammapapillomaviruses warrant further investigation.

### Accession number(s).

The GenBank accession numbers for the HPV-219, HPV-220, HPV-221, and HPV-222 genome sequences are MH172376, MH172377, MH172378, and MH172379, respectively.
